# Mpox Perceptions and Vaccine Advocacy among the Healthcare Workers of Solid Organ Transplant Centers: A Multicenter, Cross-Sectional Survey in Saudi Arabia

**DOI:** 10.3390/healthcare11040603

**Published:** 2023-02-17

**Authors:** Khalid Alhasan, Malik Sallam, Fadi Aljamaan, Tariq Ali, Ahmed Al-jedai, Ahmed Nazmi, Aziza Ajlan, Hassan Aleid, Enaam Karar, Moheeb Al-Awwami, Hamad Almojalli, Yaser Zahir Shah, Amir Eltayeb Ismail Mohammed, Mazin Barry, Sarah Alsubaie, Abdulrahman Altheaby, Reem S. Almaghrabi, Sumayah Askandarani, Ziad A Memish, Jaffar A. Al-Tawfiq, Dieter Clemens Broering, Mohamad-Hani Temsah

**Affiliations:** 1 Solid Organ Transplant Center of Excellence, King Faisal Specialist Hospital and Research Center, Riyadh 11564, Saudi Arabia; 2 College of Medicine, King Saud University, Riyadh 11362, Saudi Arabia; 3 Pediatric Department, King Saud University Medical City, King Saud University, Riyadh 11362, Saudi Arabia; 4 Department of Pathology, Microbiology, and Forensic Medicine, School of Medicine, The University of Jordan, Amman 11942, Jordan; 5 Department of Clinical Laboratories and Forensic Medicine, Jordan University Hospital, Amman 11942, Jordan; 6 Critical Care Department, King Saud University Medical City, King Saud University, Riyadh 11362, Saudi Arabia; 7 Therapeutic Affairs, Ministry of Health, Colleges of Medicine and Pharmacy, Alfaisal University, Riyadh 11533, Saudi Arabia; 8 Division of Infectious Diseases, Department of Internal Medicine, King Saud University Medical City, King Saud University, Riyadh 11362, Saudi Arabia; 9 Division of Infectious Diseases, Faculty of Medicine, University of Ottawa, Ottawa, ON K1H 8M5, Canada; 10 Organ Transplant Center, King Saud bin Abdulaziz University for Health Sciences, King Abdulaziz Medical City, Riyadh 11426, Saudi Arabia; 11 Section of Transplant Infectious Diseases, Organ Transplant Center of Excellence, King Faisal Specialist Hospital and Research Center, Riyadh 11564, Saudi Arabia; 12 Multiorgan Transplant Centre, King Fahad Specialist Hospital, Dammam 34258, Saudi Arabia; 13 Research and Innovation Center, King Saud Medical City, Ministry of Health & College of Medicine, Alfaisal University, Riyadh 11533, Saudi Arabia; 14 Hubert Department of Global Health, Emory University, Atlanta, GA 30322, USA; 15 Specialty Internal Medicine and Quality Department, Johns Hopkins Aramco Healthcare, Dhahran 34465, Saudi Arabia; 16 Infectious Disease Division, Department of Medicine, Indiana University School of Medicine, Indianapolis, IN 46202, USA; 17 Infectious Disease Division, Department of Medicine, Johns Hopkins University School of Medicine, Baltimore, MD 21218, USA

**Keywords:** monkeypox KAP, emerging infectious disease, solid organ transplant, Mpox vaccine advocacy in immune-suppressed patients

## Abstract

Background: In response to the global Mpox outbreaks, this survey aimed to assess the knowledge, perceptions, and advocacy of Mpox vaccines among solid organ transplant healthcare workers (HCWs) in Saudi Arabia. Methods: A cross-sectional survey was conducted among solid organ transplant HCWs in Saudi Arabia from 15 August to 5 September 2022. A total of 199 responses were received from participants primarily working in the kidney (54.8%) and liver (14.6%) transplant units. Results: The survey found that most participants were aware of the 2022 Mpox outbreak, but the majority were more concerned about COVID-19 than Mpox. While the majority of participants thought laboratory personnel and HCWs in direct contact with Mpox patients should receive the vaccine, less than 60% believed that all HCWs should be vaccinated. Additionally, over half of the participants lacked knowledge of animal–human transmission of the virus. Conclusion: The results highlight the need for increased education on Mpox among transplant HCWs in Saudi Arabia, particularly regarding the virus’s transmission dynamics and vaccines. This education is crucial to improve HCWs’ understanding of this emerging disease, especially given their vulnerability during the COVID-19 pandemic.

## 1. Introduction

The World Health Organization (WHO) declared the monkeypox (Mpox) outbreak to be a public health emergency of international concern (PHEIC) on 23 July 2022 [1]. On 28 November 2022, the WHO recommended using the name Mpox as a new name for the disease to reduce the associated stigma [2].

As of 18 January 2023, a cumulative total of 84,901 laboratory-confirmed cases of Mpox, including 83 deaths, have been reported from 110 countries spanning the six WHO regions [3].

The ten countries that reported the most cumulative cases worldwide are the United States of America (USA, n = 30,026), Brazil (n = 10,671), Spain (n = 7513), France (n = 4114), Colombia (n = 4062), the United Kingdom (UK, n = 3730), Peru (n = 3711), Mexico (n = 3696), Germany (n = 3684), and Canada (n = 1460) [3]. Combined, these countries represent 86% of reported cases worldwide [3].

Mpox is caused by the monkeypox virus (MPXV), a member of the *Orthopoxvirus* genus in the family *Poxviridae*, which refers to the first isolation of captive monkeys sent to Denmark from Africa in 1958, which was first identified in humans in 1970 in the Democratic Republic of the Congo [4,5]. The MPXV has a zoonotic origin, with multiple reservoirs [4]. Mpox is a disease of global public health importance as it affects not only countries in west and central Africa, but the rest of the world [4].

Mpox is transmitted to humans through close contact with an infected person or animal or with material contaminated with the virus [4].

There has been a significant increase in the recent resurgence of Mpox, which is worrying about the possibility of developing another pandemic similar to that of COVID-19 [5,6]. The recent outbreak of Mpox in several countries, with no movement to endemic regions, is certainly of worry [5,6], as demonstrated by scientists worldwide [7].

The recipients of solid organ transplantation may contract Mpox through direct contact with cutaneous lesions, sexual transmission, or respiratory droplets from infected humans or animals [8,9]. The American Society of Transplantation (AST) announced that although there were no published data on Mpox in transplant recipients, there was an impending risk to this immunocompromised patient population, especially if the human-to-human transmission continued [10].

Moreover, it could be transmitted from an infected donor to the recipient of the solid organ during transplantation [11]. The emergence of Mpox continues to add to the current burden of anxiety experienced by healthcare workers (HCWs) and the public as well [1]. A recent study revealed that approximately 62% of the general population were more worried about Mpox than coronavirus disease 2019 (COVID-19) [12]. Another study by Gallè et al. showed that the communication about Mpox was initially ineffective in terms of disease knowledge among adults [13]. The ongoing COVID-19 pandemic has been associated with stress among HCWs, as well as increased workload and anxiety [14].

The transplant community is concerned about the potential for more severe outcomes in transplant recipients if they develop Mpox infections. Moreover, transplant healthcare workers are worried about the need for post-exposure prophylaxis for themselves. We conducted this multicenter survey to assess the perceptions and knowledge among solid organ transplants’ HCWs working in Saudi Arabia and their advocacy of the Mpox vaccines.

## 2. Materials and Methods

### 2.1. Study Design and Data Collection

We conducted the cross-sectional, electronic survey among all SOT HCWs throughout the Kingdom of Saudi Arabia (KSA) from 15 August 2022 to 5 September 2022. Participants were invited by whole sampling techniques, either through SMS or the instant messaging service WhatsApp, as both are widely used among HCWs in KSA. The HCWs were invited to complete the electronic questionnaire through the SurveyMonkey© platform, with each response allowed once from each unique IP address to ensure single entries. The first page of the survey included the IRB approval and consent to participate, explaining the research objectives, voluntary participation, and complete confidentiality. The research team followed the HCWs’ responses from their sites, with two reminders, to overcome possible lower response rates, as the literature reported survey fatigue after the COVID-19 pandemic [15].

### 2.2. Ethical Approval

The ethical approval for the current study was granted by the institutional review board (IRB) at King Saud University (22/0416/IRB) before data collection began.

### 2.3. Survey Instrument

The survey tool was modified from our previously published research on COVID-19 with specific points related to the new Mpox outbreak [16,17,18,19,20]. The final version was checked for content validity by our allocated research experts and Spiloted among 12 HCWs for clarity and consistency, with subsequent minor modifications based on the experts’ recommendations. Our research team endorsed the final version of the survey for language clarity, accuracy, and content validity.

Variables surveyed included HCWs’ sociodemographic and job-related characteristics, type of SOT service, previous COVID-19 infection status, and advocacy for Mpox vaccination. We utilized multiple questions about participants’ knowledge related to Mpox and MPXV in terms of transmission, vaccination, and required isolation precautions (Table A1 in Appendix A). Moreover, the self-reported generalized anxiety disorder (GAD7) score was used as a measure of HCW’s anxiety [21,22]. We then assessed the independent variables associated with the attitude to seek more information about Mpox and the variables associated with knowledge score.

### 2.4. Statistical Analysis

Means and standard deviations were used to describe continuous variables, frequencies, and percentages for categorically measured variables. The histogram and the Kolmogorov–Smirnov test were applied to test the assumption of normality, and Levene’s test was used to test the homogeneity of statistical variance assumption. The multi-response dichotomies analysis was used to describe the measured questions with more than one option. Cronbach’s alpha test was used to assess the internal consistency of the measured questionnaires. Multivariate binary logistic regression analysis was used to assess the variables’ independent correlation. The association between predictors with the categorically measured variables was assessed with multivariate logistic binary regression analysis, which was expressed with adjusted odds ratios (aOR) with their associated 95% confidence intervals. The beta coefficient was used to assess variables’ independent associations with continuous variables. The SPSS IBM statistical analysis program was used for statistical data analysis. The statistical alpha significance level was considered at 0.050 level.

## 3. Results

### Characteristics of the Study Participants

As the research team from all the transplant centers estimated the number of SOT HCWs working in Saudi Arabia to be around 250, the whole sample was invited to participate (with two reminders). The number of complete responses was 199, giving a response rate of 79.6%. One hundred and ninety-nine HCWs working at SOT centers in Saudi Arabia participated in the survey. Table 1 displays their sociodemographic and professional characteristics. The majority were female (62.8%). Their age is distributed almost evenly across the age groups from 25 to 54 years or older. The majority were married or ever married (77.9%). Regarding their clinical role, the majority were nurses or transplant nurse coordinators 49.7%, 21.6% were consultants or associate consultants, 14.6% were assistant consultants or in-training fellows’ physicians, 8% were transplant clinical pharmacists, and 6% were immunology Lab technicians. Most of the participants worked at Kidney Transplant Units 54.8%, 14.6% worked at Liver Transplant Units, 2.5% worked at Lung/Heart transplant units, and 28.1% worked at multiorgan solid transplant units. Of the participants, 58.8% were previously diagnosed with COVID-19.

**Figure 1:**
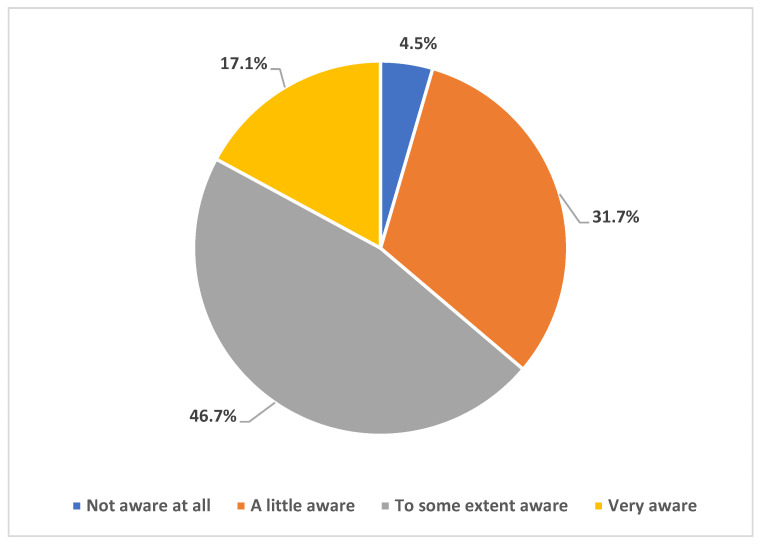
Participants’ awareness of the recent increase in Mpox cases worldwide.

**Figure 2:**
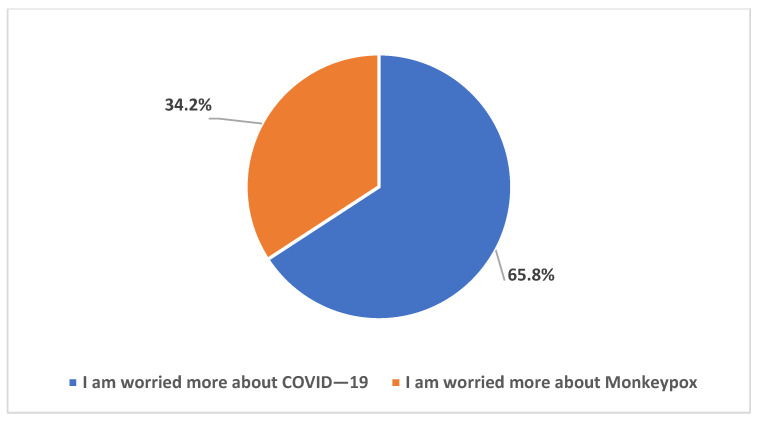
Participants’ worry about Mpox compared to COVID-19.

To explore our participants’ knowledge of monkeypox disease, we assessed their awareness of the recent outbreaks of the disease worldwide. As shown in Figure 1, 46.7% indicated they are to some extent aware, while 31.7% were just a little aware, and only 17.1% very aware. Figure 2 dissects the participants’ worries about Mpox disease as compared to COVID-19. The majority (65.8%) were more worried about COVID-19.

In relation to vaccination, we assessed the participants’ perception of Mpox vaccine administration priority apart from SOT recipients, and 87.3% of the surveyed participants perceived that laboratory personnel working directly with MPXV and HCWs caring for Mpox infected/suspected patients are the highest priority to receive the vaccine. In comparison, 59.4% perceived all HCWs in general as candidates for vaccination. Table 2 presents the details of the participants’ prioritization.

**Figure 3:**
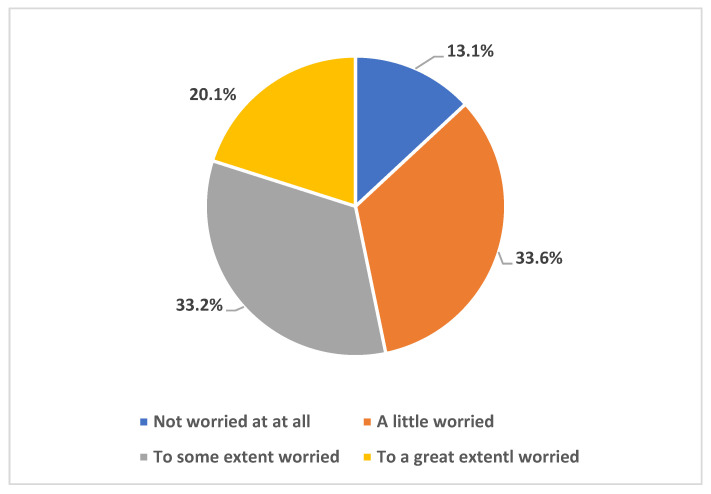
Participants’ worry level of Mpox causing worldwide pandemic such as COVID-19.

**Figure 4:**
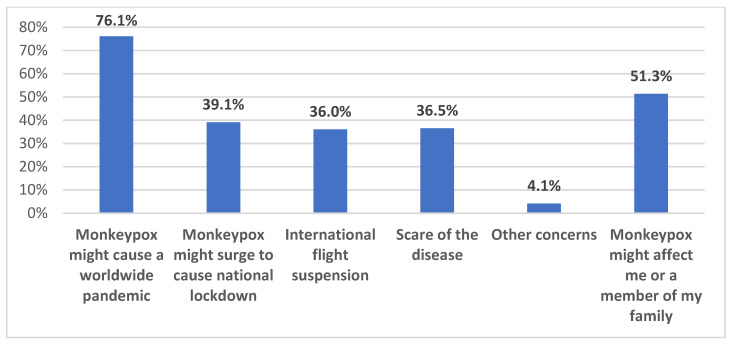
Participants’ sources of worry of Mpox disease.

Regarding the participants’ worry of the current monkeypox disease outbreak progressing to a worldwide pandemic similar to COVID-19, 33.6% were a little worried, 33.2% were worried to some extent worried, while 20.1% were worried to great extent as shown in Figure 3. When considering their sources of worry, 51.3% were worried about themselves or their families becoming infected with the virus, the majority (76.1%) were worried about monkeypox to progressing to a worldwide pandemic, 39.1% were worried about it causing national lockdown similar to the COVID-19 disease, and 36% were worried about international flight suspension 36%. Their worries are shown in Figure 4.

**Figure 5:**
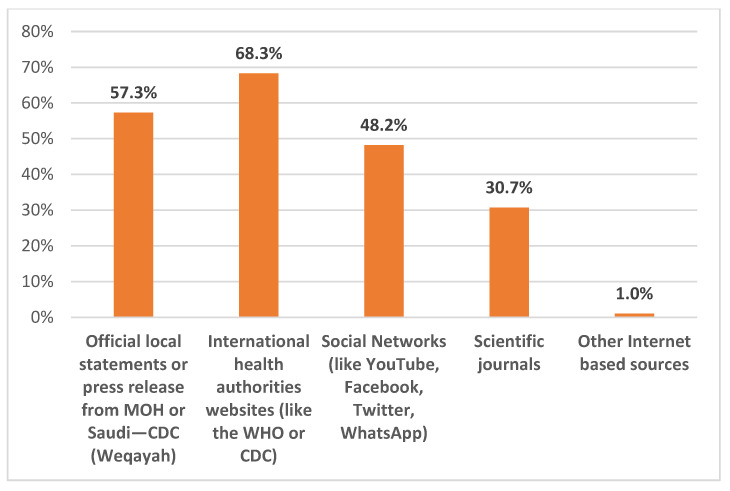
Participants’ sources of information about Mpox disease.

The healthcare workers’ sources of information and updates about Mpox disease were as follows: 57.3% relied on local MOH website for released information, 68.3% relied on international health websites, such as the WHO and Center for Disease Control and prevention (CDC), another 48.2% of the workers used social media information, and 30.7% relied on scientific journal information as sources of Mpox disease information (Figure 5).

We assessed the participants’ Monkeypox disease knowledge using four domains: clinical presentation with 13 questions, transmission modes 7 questions, precautionary measures 4 questions, and vaccination 7 questions. Table A1 in Appendix A shows the correct answers according to our expert panel and the participants’ answers.

**Figure 6:**
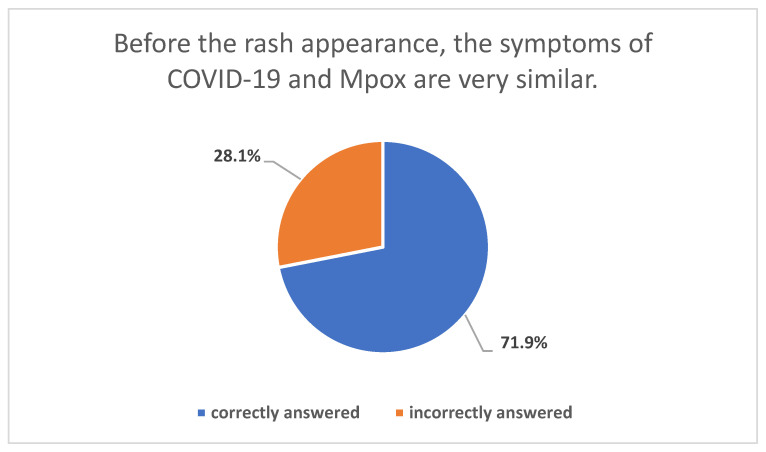
Participants’ perceptions about the similar symptoms of COVID-19 and Mpox.

Table 3 displays the participant’s overall monkeypox disease knowledge score and its different domains score analysis. The overall mean knowledge score was 20.35/32. The highest domain score was achieved in the monkeypox disease precautionary isolation measures (2.94/4), followed by transmission modes knowledge (5.8/8), then clinical presentation knowledge score (9.41/13), while the lowest score was achieved in the Monkeypox disease vaccine knowledge (2.19/7). Most participants (71.9%) correctly identified that both Mpox and COVID-19 may present similarly before the rash appearance (Figure 6).

Table 4 explains the bivariate correlation between participant’s overall Mpox disease knowledge score and their other measured perceptions. The knowledge score correlated significantly but weakly and positively with their awareness about the recent outbreaks of Mpox disease (rho = 0.146, *p*-value < 0.050), while it did not correlate with their worry about the Mpox causing a pandemic similar to COVID-19. On the other hand, their worry about Mpox disease causing a pandemic correlated significantly and positively but weakly with their GAD7 score (rho = 0.155, *p*-value = 0.050). Logically, their awareness of the recent outbreaks of Mpox disease corelated positively and with high significance with their worry about Mpox disease causing a pandemic (rho = 0.266, *p*-value < 0.010).

We looked at the participants’ predictors associated with their overall Mpox disease knowledge score as shown in Table 5 using multivariable general linear models analysis. Clinical role did not converge with knowledge score with any significance. Participants who believed that HCW’s and Lab technicians in direct contact with Mpox specimens or highly suspected patients should be prioritized for Mpox vaccines had significantly higher overall knowledge score (11.2%, *p*-value < 0.001). Considering participants’ source of information, those who relied on the international health websites (WHO and CDC) had significantly higher scores (6.2% times, *p*-value = 0.003), as did those who relied on scientific journals (7.5% times higher, *p*-value < 0.001). Regarding sociodemographic characteristics, those who reported that they ever married had slightly significant lower scores (5.1% times less, *p*-value = 0.035).

The participants’ overall Mpox vaccine knowledge showed that their overall Mpox disease knowledge score had correlated positively and significantly with their vaccine knowledge score (RR 1.120, *p* value < 0.001). When considering participants’ clinical role, nurses and transplant nurse coordinators had a significantly lower vaccine knowledge score compared to others (36.8% times less, <0.001. All other participants’ measured predictor variables did not correlate significantly with their Mpox vaccines knowledge score when tested with other iterative models (Table 6).

As Mpox clinical presentation can mimic COVID-19 initially before the appearance of the skin rash, and with the latter still circulating in most communities at the time of data collection, we explored the participants’ awareness of this potential diagnostic dilemma and its potential contributing factors. Only 52.3% correctly knew this potential diagnostic challenge. As shown in Table 7, assistant consultants/in training fellows were significantly less likely (62%, *p*-value = 0.031) to be aware of this similarity. Moreover, participants’ overall Mpox knowledge had correlated significantly and negatively with their odds of being aware of such similarity of initial presentation (OR 0.849, *p*-value = 0.002).

## 4. Discussion

The recipients of SOT are among the groups considered at risk for MPXV acquisition with risk of severe disease. This comes in relation to their compromised immune status; therefore, HCWs involved in SOT patient care should be highly knowledgeable regarding this emerging infection.

The current study represented a unique and novel opportunity to analyze Mpox knowledge and awareness as well as the worries of this group towards an emerging infection that was declared as a PHEIC.

The findings of the study indicated several gaps in HCWs’ knowledge and relatively high levels of anxiety regarding Mpox among the study group. The relevance of the study is related to the representativeness of various occupational categories of HCWs involved in SOT and the inclusions of personnel with varying level of experience. Specifically, about half of the study sample comprised nurses or nurse coordinators. Moreover, more than a third of the respondents were physicians. Furthermore, the study sample comprised pharmacists and laboratory technicians having different roles in the care of SOT patients.

Our results indicated high levels of awareness of the ongoing Mpox multi-country outbreak, with >95% of the participants being either very aware, aware, or at least having a little awareness of the 2022 increase in Mpox cases worldwide that meant its declaration as a PHEIC. This result is conceivable for two reasons: first, the SOT HCWs could have higher levels of worry regarding emergence infectious diseases considering the critical condition of the patients they take care of; second, the timing of the survey (August/September 2022) coincided with intensive and rapid availability of literature regarding Mpox together with intensified media coverage of the new emerging infection following COVID-19. The latest point might explain the finding of high levels of worries that Mpox might turn into a pandemic similar to COVID-19. Specifically, 87% of the respondents were either very worried, worried to some extent, or at least having a little worry that MPXV will cause a pandemic similar to COVID-19. This result is much higher compared to the level of worry previously observed among HCWs in Saudi Arabia, where the level of Mpox worries was observed at a rate of 51% [23]. This high level of worry among SOT HCWs is also understandable considering the immune status of the patients to whom they are responsible for providing care. However, the respondents listed other possible causes of such worries including the fear of another pandemic, fear of becoming infected themselves or infection among their families, and worries that Mpox might cause another international flight suspension or wide lockdowns. Despite that, the level of worry from COVID-19 was still higher among the study respondents compared to the level of worry from Mpox (66% vs. 34%). In line with this result, the overall level of anxiety as measured through the GAD-7 score revealed a mean score of 3.6 out of 21 maximum points.

Regarding the level of Mpox knowledge, variable defects were observed as follows: first, the most severe gaps in Mpox knowledge were observed for the items assessing prevention through vaccination. Specifically, correct knowledge of the recommended vaccine for SOT patients (JYNNEOS) was only found among less than a quarter of the respondents. Additionally, correct knowledge of the safety of the MVA vaccine among SOT patient was found among merely 10% of the study sample. Furthermore, 79% of the participants incorrectly thought that chickenpox vaccination can be protective against Mpox. Previous studies reflected that inadequate Mpox vaccine knowledge is commonplace in various studies worldwide. For example, an early survey among Saudi physicians found that the awareness of availability of vaccines to prevent Mpox was reported at a rate of 70% [24]. Much lower rate of Mpox vaccine availability was observed among HCWs in Jordan, where less than a third of the participants had such knowledge [25]. Improving the level of vaccine knowledge is of particular importance among SOT HCWs considering the previous evidence that better knowledge can be linked with a favorable attitude towards vaccination and, in turn, a higher likelihood of recommending vaccination to the SOT patients who are considered at a higher risk of severe disease [26,27].

Second, the level of Mpox knowledge regarding the possible transmission route was slightly better. However, defects in knowledge were observed for lack of transmission through respiratory droplets, with only 47% correct responses, and regarding sexual transmission of MPXV, with only 57% correct responses. Similarly, inadequate knowledge of Mpox transmission was observed among Italian and Indonesian physicians, as well as HCWs in Kuwait, Jordan, and the Czech Republic [25,27,28,29,30]. Moreover, in a recent review of 16 studies that were conducted in non-endemic countries, there were unsatisfactory levels of knowledge and awareness among both HCWs and the general adult population [31].

Third, variable knowledge was observed for the items assessing the initial clinical presentation of Mpox as follows: knowledge of fever and skin rash was found in the vast majority of the participants (>90%). Nevertheless, inadequate knowledge of the following signs/symptoms was found among the participants: lymphadenopathy (53%); exhaustion (37%). Finally, several areas need improvement regarding the precautionary measures needed to halt Mpox spread, where 22% and 27% of the participants were not aware of the importance of contact and airborne precautions, respectively.

From a broader perspective, the overall Mpox knowledge was evaluated at a rate of 20.4 out of 32 points as a maximum score. This highlights the need for educational efforts to address these gaps in SOT HCWs’ knowledge. The importance of educational efforts needed to increase the confidence of HCWs to diagnose and manage Mpox case and subsequently mitigate the spread of the virus was underlined in a recent review by Di Gennaro et al. [32]. The previous and recent studies showing inadequate Mpox knowledge highlights the importance of educational intervention measures that can improve the attitude towards the patients as well, which was advocated by the WHO to limit Mpox spread [24,25,27,29,33,34,35,36,37].

In this study, a special aspect of Mpox knowledge evaluation was related to the finding of lower Mpox vaccination knowledge among nurses and nurse coordinators in multivariate analysis. This recurring pattern was also found in recent studies among HCWs in Jordan and Kuwait [25,28], highlighting the need for educational programs tailored to meet the needs of different occupational categories of health professionals.

Treatment of Mpox in immune-compromised patients could be optimized if initiated early and in accordance with the best available evidence. At the time of our research, there were no approved specific antivirals for Mpox therapy and the mainstay relied on supportive treatment. In the Saudi ministry of health guidelines, the two suggested medications were Brincidofovir (once available) and Vaccinia immune globulin (SPIG) (for severe cases) [38]. In a meta-analysis of 71 individuals, the most commonly used antiviral was tecovirimat, and cidofovir was used in seven patients and brincidofovir was used for three patients [39].

An important area to be considered in the awareness and educational programs is the source of Mpox information. In this study, the majority of respondents reported the reliance on trusted sources of information (e.g., the WHO and CDC websites, the MOH website). However, it was noteworthy to find that almost half of the participants used social networks as a source of updates regarding Mpox. This should be considered carefully, considering the previous evidence of the easy spread of misinformation regarding infectious diseases through social media outlets, which was noticeable during the COVID-19 pandemic [40,41]. In line with this concept, conspiracy beliefs regarding Mpox among emerging infections were noticeable from the early days of the Mpox outbreak declaration [25,28,36,42]. The importance of the source of Mpox information was revealed in our results; we found better Mpox knowledge in the multivariate analysis if the source of information was based on scientific journals or the international health authorities’ websites (the WHO, CDC).

### Strengths and Limitations

Our study is the first study to explore the solid organ transplant HCWs’ Mpox perceptions and vaccine advocacy. In the face of the emerging alert of an infectious disease that is novel to the local healthcare system, we were able to identify areas of improvement in Mpox awareness and vaccine advocacy among the HCWs for this vulnerable patient population. Limitations of our research may relate to our inability to explore the HCW’s previous travel experience to countries with endemic Mpox. Other limitations relate to cross-sectional survey limitations in relation to the sampling technique and recall bias.

## 5. Conclusions

HCWs working in transplant require further education about the Mpox disease and its transmission dynamics and require support. This is particularly important as HCWs showed significant vulnerability during the COVID-19 pandemic. Further studies to elucidate factors associated with HCW worries and anxiety are warranted.

## Figures and Tables

**Table 1:** Descriptive analysis of the HCW’s sociodemographic (n = 199).

Variable	Number	Percentage
**Sex**		
Female	125	62.8
Male	74	37.2
**Age group**		
25–34 years	66	33.2
35–44 years	77	38.7
45–54 years and older	56	28.1
**Marital state**		
Never married	44	22.1
Ever married	155	77.9
**Clinical Role**		
Consultant/Associate consultant	43	21.6
Assistant consultant/In-training fellow	29	14.6
Transplant Clinical Pharmacist	16	8.0
Nurses/ Transplant nurse Coordinators	99	49.7
Immunology Laboratory Technician	12	6.0
**Participants’ organ transplant involvement**		
Kidney	109	54.8
Liver	29	14.6
Lung/Heart	5	2.5
Multi-Organ	56	28.1
**Participants’ COVID-19 infection status**		
No	82	41.2
Yes	117	58.8

**Table 2:** Participants’ prioritization of Monkeypox disease vaccine administration.

Item	Number	Frequency
Healthcare professionals	117	59.4
High-risk HCWs #	172	87.3
College students	26	13.2
Pregnant ladies	56	28.4
Children	38	19.3
Other	2	1.0

Mpox: monkeypox; HCW: healthcare worker; COVID-19: coronavirus disease 2019; SOT: solid organ transplantation; MPXV: Mpox virus; #: Laboratory personnel working directly with MPXV and HCWs caring Mpox infected/suspected patients.

**Table 3:** Participants’ Mpox disease knowledge scores.

Aspect	Mean	SD	Median	IQR	Maximum Possible Score
Knowledge score of Mpox transmission mode	5.80	1.11	6.0	2.0	0–8 points
Knowledge score of Mpox vaccines	2.19	1.40	2.0	1.0	0–7 points
Knowledge of Mpox presenting signs and symptoms score	9.41	1.63	9.0	3.0	0–13 points
Knowledge of Mpox precautionary-isolation methods score	2.94	0.90	3.0	1.0	0–4 points
Overall Mpox knowledge score	20.35	3.10	20.0	5.0	0–32 points
Generalized Anxiety (GAD-7) score	3.61	4.70	2.0	5.0	0–21 points

**Table 4:** Bivariate Spearman’s correlations (Rho) test between the HCW’s measured perceptions and knowledge about Mpox.

Variable	Knowledge Mpox	GAD7	Awareness Mpox
Total Mpox knowledge score	1.000		
Generalized Anxiety GAD-7 score	0.100		
Participants’ awareness about the recent Mpox outbreaks	0.146 *	0.077	
Participants’ worry of Mpox causing pandemic similar to COVID-19	0.099	0.155 *	0.266 **

* Correlation is significant at 0.050 level, ** correlation is significant at 0.010 level.

**Table 5:** Multivariate general linear regression gamma analysis of the participants’ variables associated with Mpox disease overall knowledge score of (n = 199).

Parameter	Multivariate Adjusted Risk Rate	95% Wald CI for RR		
		Lower	Upper	*p*-Value
(Intercept)	16.526	15.050	18.146	<0.001
Sex = Male	1.030	0.986	1.075	0.184
Age	1.021	0.995	1.047	0.115
Marital state = Ever married	0.949	0.904	0.996	0.035
Profession= Nurses and Nurse Transplant coordinators	0.962	0.923	1.004	0.073
Mpox vaccination priority #	1.117	1.058	1.180	<0.001
Believes pregnant women should be prioritized for vaccination against Mpox.	0.965	0.926	1.005	0.087
Source of information about Mpox = Local official statements (MOH)	1.035	0.996	1.075	0.076
Source of information about Mpox = International health authorities websites (such as the WHO or CDC)	1.062	1.020	1.106	0.003
Source of information about Mpox = Scientific journals	1.075	1.032	1.120	<0.001
General awareness of Monkeypox global recent outbreaks	1.014	0.989	1.038	0.277
Generalized Anxiety GAD-7 score	1.002	0.998	1.006	0.444

Dependent variable: overall Mpox knowledge score. # Believes that laboratory personnel working directly with MPXV and HCWs caring Mpox infected/suspected patients should be prioritized for Mpox vaccines.

**Table 6:** Multivariate general Linear regression gamma analysis of the participants’ variables associated with their overall Mpox vaccines knowledge (n = 199).

Parameter	Multivariate Adjusted Risk Rate	95% Wald CI for RR		
		Lower	Upper	*p*-Value
(Intercept)	0.276	0.117	0.651	0.003
Sex = Male	0.907	0.698	1.178	0.463
Age group	0.897	0.767	1.049	0.173
Marital state = ever married	1.234	0.925	1.645	0.153
Mpox overall disease knowledge score	1.120	1.078	1.164	<0.001
Generalized Anxiety GAD-7 score	1.004	0.980	1.029	0.762
Profession = Nurses & nurse transplant coordinators	0.632	0.493	0.811	<0.001

Dependent variable: participants’ overall Mpox vaccine knowledge.

**Table 7:** Multivariable binary logistic regression of variables associated with participants’ awareness of Mpox and COVID-19 clinical presentation (n = 199).

Parameter	Multivariate Adjusted Odds Ratio	95% Wald CI for OR		
		Lower	Upper	*p*-Value
(Intercept)	73.421	6.930	777.921	<0.001
Sex = Male	1.293	0.680	2.459	0.434
Age group	0.890	0.602	1.316	0.560
Profession = Assistant consultant	0.380	0.157	0.916	0.031
Worry level of possible spread of Mpox becoming pandemic	0.741	0.539	1.019	0.065
Overall knowledge of Mpox disease	0.849	0.765	0.943	0.002

Dependent variable: believes Mpox can mimic COVID-19 initially before the appearance of the skin rash.

## Data Availability

Data is available upon reasonable request from the corresponding author.

## Appendix A

**Table A1:** Descriptive analysis of the healthcare workers knowledge about various Mpox disease aspects.

Mpox Knowledge Item	Correct Answer	Incorrectly Answered	Correctly Answered
**Knowledge of Mpox ^1^ vaccines**			
SOT ^2^ patients should receive any available Mpox vaccine as soon as possible	**False**	62 (31.2)	137 (68.8)
SOT patients should receive the MVA ^3^ vaccine (available as JYNNEOS in the U.S.) is recommended as post-exposure prophylaxis	**True**	151 (75.9)	48 (24.1)
The smallpox vaccine, ACAM2000, is a live replicating virus and is contraindicated in SOT and immunocompromised patients	**True**	145 (72.9)	54 (27.1)
MVA is a live attenuated virus vaccine, but it cannot replicate in human cells, and is considered safe in SOT recipients	**True**	179 (89.9)	20 (10.1)
Data are limited to support the efficacy of MVA and VIGIV ^4^ against the current Mpox.	**True**	112 (56.3)	87 (43.7)
HCWs ^5^ exposed to a case of Mpox should receive PEP ^6^ with the smallpox vaccine	**True**	177 (88.9)	22 (11.1)
Chickenpox vaccine, such as Varivax, has dual activity against Chickenpox and Mpox.	**False**	157 (78.9)	42 (21.1)
**Knowledge of Mpox modes of transmission**			
Animal-to-human	**True**	114 (57.3)	85 (42.7)
Human-to-human via direct skin contact	**True**	53 (26.6)	146 (73.4)
Human-to-human via sexual route	**True**	86 (43.2)	113 (56.8)
Airborne	**False**	45 (22.6)	154 (77.4)
Droplet	**True**	105 (52.8)	94 (47.2)
Food-Borne	**False**	11 (5.5)	188 (94.5)
Contaminated water	**False**	20 (10.1)	179 (89.9)
Other modes	**False**	3 (1.5)	196 (98.5)
**Knowledge of Mpox signs and symptoms**			
Before the rash appears, the symptoms of COVID-19 ^7^ and Mpox are very similar	**True**	95 (47.7)	104 (52.3)
**Initial presentation symptoms/signs**			
Fever	**True**	19 (9.5)	180 (90.5)
Rash	**True**	16 (8.0)	183 (92.0)
Headache	**True**	64 (32.2)	135 (67.8)
Lymphadenopathy	**True**	94 (47.2)	105 (52.8)
Myalgia	**True**	87 (43.7)	112 (56.3)
Exhaustion	**True**	126 (63.3)	73 (36.7)
Respiratory distress	**False**	64 (32.3)	135 (67.8)
Shock-hemodynamic instability	**False**	15 (7.5)	184 (92.5)
Seizure	**False**	17 (8.5)	182 (91.5)
Loss of the sense of smelling	**False**	11 (5.5)	188 (94.5)
Acute kidney injury	**False**	17 (8.5)	182 (91.5)
In SOT, as compared to smallpox, Mpox is causing a more severe disease	**True**	137 (68.6)	62 (31.2)
**Knowledge of Mpox isolation and precautionary measures**			
Contact precautions	**True**	44 (22.1)	155 (77.9)
Airborne precautions	**False**	54 (27.1)	145 (72.9)
Droplet precautions	**True**	109 (54.8)	90 (45.2)
Other isolation techniques	**False**	2 (1.0)	197 (99.0)

^1^ Mpox: monkeypox; ^2^ SOT: solid organ transplantation; ^3^ MVA: modified vaccinia Ankara; ^4^ VIGI: vaccinia Immune globulin Intravenous; ^5^ HCWs: healthcare workers; ^6^ PEP: post-exposure prophylaxis; ^7^ COVID-19: coronavirus disease 2019.
